# An improved approach for the segmentation of starch granules in microscopic images

**DOI:** 10.1186/1471-2164-11-S2-S13

**Published:** 2010-11-02

**Authors:** Shengwen Guo, Jinshan Tang, Youping Deng, Qun Xia

**Affiliations:** 1 Image Processing and Bioimaging Research Laboratory, System Research Institute & Department of Advanced Technologies, Alcorn State University, 1000 ASU Drive, Alcorn State, MS 39096, USA; 2 Rush Cancer Center, Rush University Medical Center, Chicago, IL 60612, USA; 3 Department of Agriculture-Rest, Alcorn State University, 1000 ASU Drive, Alcorn State, MS 39096, USA

## Abstract

**Background:**

Starches are the main storage polysaccharides in plants and are distributed widely throughout plants including seeds, roots, tubers, leaves, stems and so on. Currently, microscopic observation is one of the most important ways to investigate and analyze the structure of starches. The position, shape, and size of the starch granules are the main measurements for quantitative analysis. In order to obtain these measurements, segmentation of starch granules from the background is very important. However, automatic segmentation of starch granules is still a challenging task because of the limitation of imaging condition and the complex scenarios of overlapping granules.

**Results:**

We propose a novel method to segment starch granules in microscopic images. In the proposed method, we first separate starch granules from background using automatic thresholding and then roughly segment the image using watershed algorithm. In order to reduce the oversegmentation in watershed algorithm, we use the roundness of each segment, and analyze the gradient vector field to find the critical points so as to identify oversegments. After oversegments are found, we extract the features, such as the position and intensity of the oversegments, and use fuzzy c-means clustering to merge the oversegments to the objects with similar features. Experimental results demonstrate that the proposed method can alleviate oversegmentation of watershed segmentation algorithm successfully.

**Conclusions:**

We present a new scheme for starch granules segmentation. The proposed scheme aims to alleviate the oversegmentation in watershed algorithm. We use the shape information and critical points of gradient vector flow (GVF) of starch granules to identify oversegments, and use fuzzy c-mean clustering based on prior knowledge to merge these oversegments to the objects. Experimental results on twenty microscopic starch images demonstrate the effectiveness of the proposed scheme.

## Background

Starch, the complex carbohydrate, is a major component in human diet. It has also been widely used in industrial applications such as making food, health and nutrition, pharmaceutical and personal care. The natural starch is produced in chloroplasts by photosynthesis and it is usually packed into granules with a layered structure [1][2]. The shape and size of native starch granules vary among species. For example, potato starches have large round granules and their diameters are up to 100μm. Rice starch granules are the smallest of the cereal starches and are about 2μm in diameters [3]. In wheat, the situation is more complicated. Three types of granule size distributions were reported [4]. The shape and size of starch granules can affect the starch properties and functions; starch granules for industrial application usually have specific requirement in their shape and size. For instance, starch granules for the paper industrial are required to have spherical shape and a typical uniform granule size. Natural starches do not have such uniform characteristics, therefore they are needed to be modified for industrial applications. In the other hand, cultivation of a variety with specific shape and size of starch granules is an alternative way to meet this demand. Hence, the new cultivar has to be investigated and exploited. Developing a new cultivar with a certain desired size of starch granules for industrial has economical significance in the agriculture science.

Among several methods to measure the shape and size of granules, microscopic evaluation is the most convenient and relatively precise way [5]. The granule images were taken from a light microscope. The shape and size can be examined directly from the image prints. The microscopy images can be converted or directly acquired into digital data and analysis automatically. In addition, with this method, starch granules can be analyzed *in situ,* that is that starch granules do not need to be extracted from plant tissues. *In situ* data are significant for the study of starch granule development in related to other cellular component. Microscopy image analysis is a relative simple for quick checking the starch changes in plant tissue for breeders who are interested in developing new cultivars. The data from image analysis are the most precise and repeatable compared with others methods [5], but this method could be retarded by the image quality in more complex scenarios of overlapping granules. Distinguishing starch granules from background noise and identifying overlapping granules are a promising technique for quantitative analysis.

In order to analyze starch granules quantitatively, image segmentation technique is often adopted to segment the starch granules from the background. Segmentation is a challenging task due to the noise, the irregular shape of the objects, and the complicated topology, etc. For microscopic images of starch granules, many starch granules usually gather together and even overlap, which makes the segmentation more difficult. To deal with the contacted and overlapping objects, many approaches have been proposed [6][7]. Some approaches are based on deformable contours and level sets. Because of some disadvantages of the current active contour models and level sets, many improved versions have been proposed for real applications. For example, Ortiz de Solorzano et al. [6] presented a level set scheme for the segmentation of nuclei and cells. Vese et al[7]developed a novel multiphase level set framework based on Mumford and Shah model, and the method can avoid the problems of vacuum and overlapping automatically. Yan et al.[8]introduced an interaction model with repulsion and competition to segment high throughput RNAi fluorescent cellular images. However, these methods are time consuming and require many control parameters. In this paper, we develop image segmentation algorithm based on watershed. Watershed algorithm [9] considers the gradient magnitude of an image as a topographic surface. Pixels having the highest gradient magnitude correspond to the watershed lines, which are interpreted as the region boundaries. The successive flooding strategy is performed to construct a basin which represents a segment. The watershed method has many advantages. For example, it is simple, intuitive, can be parallelized, and always produces an entire partition of the image[10]. However, image segmentation based on watershed still has some drawbacks such as oversegmentation and sensitivity to noise. In this paper, we propose a novel merging scheme to solve the oversegmentation problem by using fuzzy c-means classification based on the prior knowledge and region features of starch granules.

## Methods

### Segmentation using thesholding and watershed

The rough segmentation of the image is divided into two steps. The first step is to separate starch granules from background using automatic thresholding. The second step is to use watershed algorithm to roughly segment the image.

**Figure 1:**
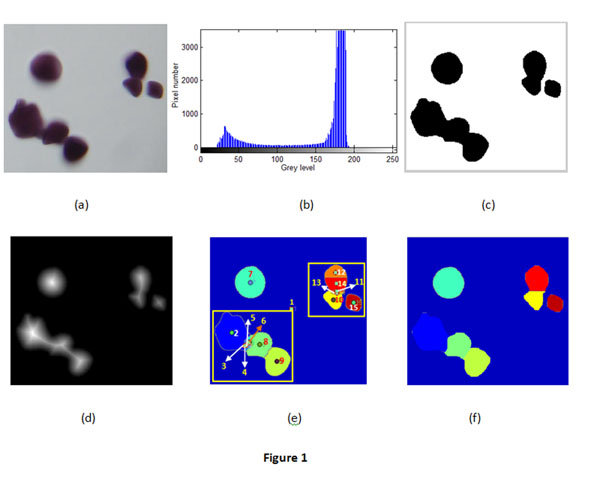
**Original image, histogram, Chamfer distance and results.** (a) Original image,(b) histogram of (a), (c) binary image using automatic thresholding (T0=109,T=115), (d) Chamfer distance ,(e) result by the watershed,(f)final result after merging by proposed method

From the microscopic images of starch granules in Fig. 1(a), we can know that the starch granules are dark with low gray values while background is light gray with high gray values. Its histogram in Fig.1 (b) shows that there are two peaks corresponding to starch granules and background respectively. Therefore it will facilitate watershed to extract the objects if starch granules can be separated from background. We employ automatic thresholding or dynamic thresholding to separate the two dominant parts. The algorithm is from [11] and is described as follow:

**Step 1.** Select an initial threshold T0 and stopping criterion ε. T0 is set as the mean of whole image, and the stopping ε is set as a very small value;

**Step 2**. Let the current threshold T equal to T0;

**Step 3.** Segment the image with threshold T as follows: If a pixel has a gray value less than T, it is classified into class C1 (starch grain part), and if the pixel has a gray value more than T, then it is classified into C2(background part).

**Step 4.** Compute the average values μ_1_ and μ_2_ for the pixels in regions C1 and C2 respectively by

 (1)

where *f*(.) is the gray values of an image, *M*, *N* are the numbers of the pixels in classes C1 and C2 respectively.

**Step 5.** Compute a new threshold value by

 (2)

Repeat step 3 through 5 until the difference in T in successive iterations is smaller than ε.

After the starch granules have been separated from background, a binary image is generated which 0 means the starch grain part and 1 means the background. After we obtain the binary image, we calculate the Chamfer distance of the binary image. Chamfer distance transformations rely on the assumption that it is possible to deduce the value of the distance at a pixel from the value of the distance at its neighbors [12][13]. Chamfer distance transforms are a class of effective discrete algorithms which offer a good approximation to the desired Euclidean distance transform which is computationally very intensive[13]. After the Chamfer distance map of the binary image is obtained, it is used as the input of watershed algorithm for object segmentation. The basic idea of watershed algorithm comes from field of topography [14]: a drop of water falling on topographical surface follows a steepest descent line until it reaches a local minimum[14]. Watershed lines are considered as divide lines to attract the drops of water. Water will fill up basins starting at these local minima and dams will be built when water coming from different basins meets [15]. The surface is partitioned into regions or basins are separated by these dams. For image segmentation, intensity gradient is usually considered as a topographic surface, each regional minimum of the gradient image is the attraction point of a catchment basin. In our algorithm, Meyer’s watershed algorithm [14] is adopted. The regions after watershed segmentation are called a region.

### Identification of oversegmentation

One of the big issues of watershed algorithm is oversegmentation when it is used for image segmentation. Oversegmentation happens when a granule is segmented into two or more segments. These segments from the same granule are called oversegments in this paper.

In order to alleviate oversegmentation, we develop a hybrid algorithm to reduce it. The proposed algorithm for reducing oversegmentation is divided into two stages. The first stage is to identify the oversegments and the second stage is to merge the oversegments to the objects which they belongs to. In the first stage, we use both shape information and gradient vector flow to identify the oversegments automatically.

Since the shape of a starch granule is round or nearly round, the roundness of an oversegment should likely be small because it’s only one part of a round object. Thus, we use the roundness values of segments to identify oversegments. The roundness is computed as

 (3)

where S and L are the area and perimeter of a segment respectively. If a segment has a roundness value less than some threshold, it is classified as an oversegment.

However, the use of roundness values of the starch granules is not enough to identify all the oversegments because some oversegments may have big roundness values. In order to alleviate this difficulty, we use the critical points of the GVF (gradient vector flow) field of segments [16] to further identify the oversegments which have not been identified using the roundness criteria. The proposed method is based on the fact that each granule should have a critical point. We consider a segment to be an oversegment if there is no critical point inside it. In order to use this method, we need to compute the GVF field of the image and then use it to compute the critical points of the objects. The GVF used in this paper is obtained using the method developed in [17][18]. Let GVF be defined as the vector field **v**(*x*, *y*) = (*u*(*x*, *y*), *v*(*x*, *y*)), then it can be obtained by minimizing the following energy functional[17][18]

 (4)

To solve the above GVF functional, the following Euler equations can be obtained using calculus of variations [17][18]

 (5)

 (6)

where ∇^2^ is the Laplacian operator. The GVF field can be obtained by treating *u* and *v* as a function of time *t*[17][18]

 (7)

 (8)

**Figure 2:**
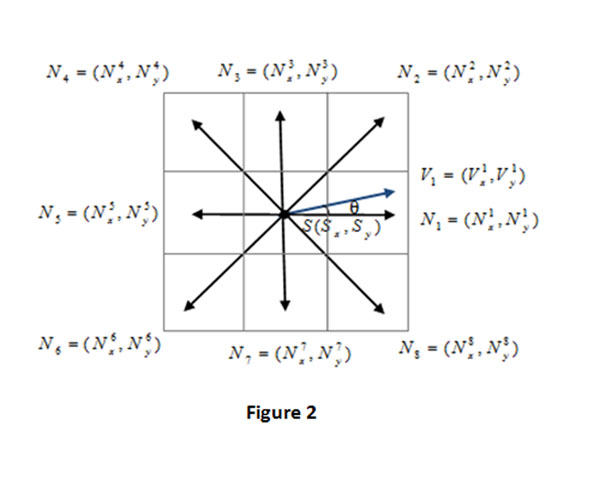
**8-neighbour directions for searching critical points and vector cosine similarity.** The ideal critical point should have a GVF force pointing outward in its 8-neighbourhood.The cosine similarity defines the cosine angle between the vectors, with values between 0 and 1.

After the GVF field is obtained, we use it to find the critical points in the segments, which are obtained by watershed segmentation. Because each object has a center, thus we will find the critical points around the center. Based on the research in [16], the 8-neighbourhood gradient vectors of an critical point should point to eight different directions outward and the critical point should has zero gradient vector in the GVF field as shown in Fig.2.

Let *S =* (*S_x_*, *S_y_*) be a candidate of a critical point,  be the *i*-th pixel in the 8-neighbourhood of pixel  be the gradient vectors obtained at pixel *N_i_*(*i* = 1,2…,8). In order to determine whether *S* is a critical point, we compute the cosine similarity between two directions, which is defined as[19][20]:

 (9)

where • is dot product and, || || denotes magnitude of a vector,  is the directional vector pointing from *S* to *N_i_*,which is obtained by

 (10)

The cosine similarity is from 0 to 1, and bigger cosine similarity means higher similarity of two directions as in Fig.2. In our experiments, if the similarity value between two directions is more than 0.90, it can be considered similar enough or the same. A critical point can be detected if there are more than 6 directions similar enough to the 8-neighborhood directions as in Fig.2. If the segment doesn’t have critical point, then it is identified as an oversegment.

After roundness criterion and critical point criterion are used to identify the oversegments, there are still some oversegments which are not identified. In the experiments, we found that an oversegment which is not identified by the two criteria is the segment which occupies more than half of an object. Thus, after identification processing, the segments are classified into three types: (1) oversegments identified; (2)oversegments unidentified; (3) single objects. We call the second and the third types of segments core segments because they are segments with big round values and critical points.

### Merging oversegments

After the oversegments are detected, the fuzzy c-mean classifier is applied to merge them. In classical cluster analysis, each datum must be assigned to exactly one cluster [17]. Fuzzy cluster analysis relaxes this requirement by allowing the gradual memberships to improve the efficiency and robustness of classification. The most widely used clustering method is FCM(fuzzy c-means) [21], which allows the number of clusters to be automatically adjusted during the iteration by merging similar clusters and splitting clusters with large standard deviations. Given a set of data pattern, , the algorithm tries to minimize the weighted within group sum of squared error object function *J(U,V)*[21]

 (11)

where *x_k_* is the *k*-th *p*-dimensional data vector, *v_i_* is the center of class *i*, *u_ik_* is the degree of membership of *x_k_* in the *i-th* class, *m* is the weighting exponent on each fuzzy membership, *d*(*x_k_,v_i_*) is the distance between data pattern *x_k_* and the cluster center *v_i_*, *n* is the number of data and *c* is the number of clusters. Here *u_ik_* satisfies

 (12)

Minimization of objective functional in (11) can be solved by an iterative process as follows[21]:

 (13)

 (14)

Fuzzy c-means is a data clustering technique based on the features of the observed objects, thus effective features selection plays a vital role in merging similar clusters and splitting clusters. Since intensity and position are the main features to distinguish different segments, the mean intensity, intensity variance are the two primary features of a segment. The space position is also important to investigate the relationship between segments. After being clustered by the watershed, the center of each segment is obtained. We can calculate the distance between segment center and the center of region of interest (ROI), which includes the supposed oversegments. In order to indicate the importance of the features, we can assign weight to each feature. The distance should be given bigger weight for it’s more important when two or more adjacent segments are determined to merge. In our experiment, the weights are 0.25, 0.25, 1.0 for mean intensity, variance of intensity and centers distance.

Our proposed method can be described as follows:

**Step 1.** Separate background and starches using automatic thresholding and obtain a binary image;

**Step 2.** Calculate Chamfer distance of the binary image;

**Step 3.** Segment objects using watershed algorithm based on Chamfer distance map;

**Step 4.** Compute the roundness of the segments by watershed method, and then identify the oversegments with small roundness;

**Step 5.** Analyze the GVF fields of segments with big roundness and search critical points so as to identify the oversegments without critical points.

**Step 6.** Extract the features for segments.

**Step 7.** Initialize cluster centers using centers of core segments.

Step 8. Merge the oversegments identified to the core segments by Fuzzy c-means clustering.

## Results

In this paper, we used sweet potato starch as an object to present a method to improve microscopy image analysis. Sweet potato starch was extracted from storage roots and diluted in water and stained with I2/IK (iodine-potassium iodide solution) . These stained starch granules were then put on microscopy slides and covered with a glass. The slides were analyzed under bright-field illumination with a microscope (Olympus). Images were captured with a high-resolution CCD color camera (DP71 Olympus). Fig.1 (a) shows a sample of a microscope image with 7 starch granules.

The first experiment was performed to segment the image roughly using thresholding and watershed algorithm. The results are shown in Fig.1. Fig.1 (b) is the histogram of the image shown in Fig.1 (a). From Fig.1 (b), we can find that the histogram has two peaks, which correspond to the gray background and the black granules respectively. The histogram shows that thesholding is possibly an effective method to separate the background from the foreground. Fig.1(c) is the binary image obtained using automatic thresholding. In the thresholding processing, the initial thresholding was set to be 109, and ε was set to be 0.2. The automatic thresholding stopped when the threshold reached 115. The segmentation result shown in Fig.1 (c) verifies that thesholding method has good performance. After the binary image was obtained using thresholding, Chamfer distance of the binary image was computed and then used as the input of the segmentation algorithm using watershed. The Chamfer distance of the binary image is shown in Fig.1(d), and Fig.1(e) shows the segmentation result obtained by watershed algorithm on Chamfer distance map in Fig.1(d). In Fig. 1(e), there are 15 segments which were filled with different colours and labelled by different numbers from 1 to 15 (background labelled with 1). From Fig. 1(e), we can find that oversegmentation happened in the segmentation by watershed algorithm.

**Figure 3:**
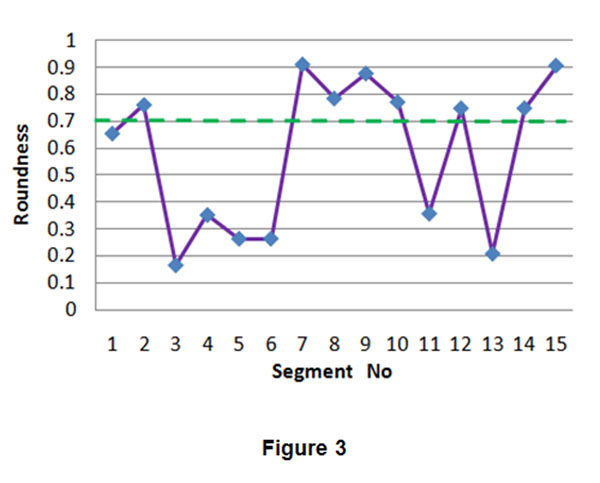
**Roundness of segments.** The threshold is 0.70, segments 1,3,4,5,6,11,13 have roundness less than 0.70, while segments 2,7,8,9,10,12,14,15 have roundness more than 0.70.

**Figure 4:**
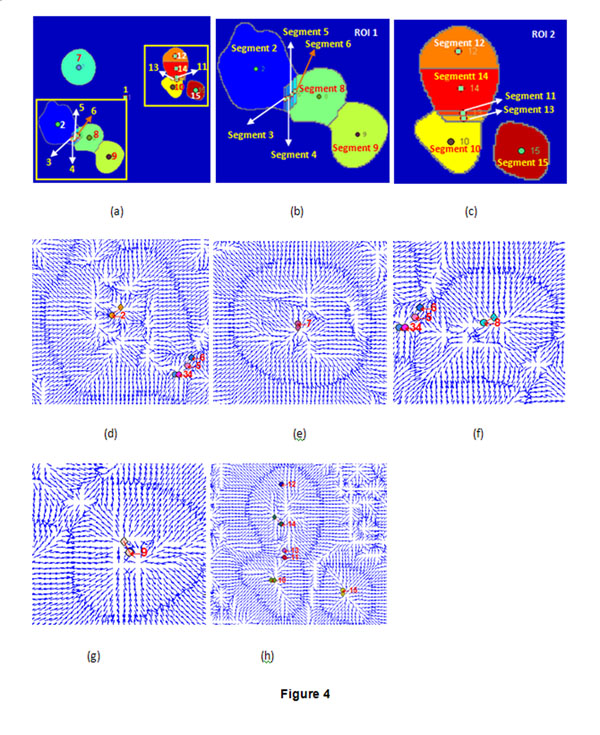
**ROIs and GVF field.** (a) Result by watershed;(b) ROI 1;(c) ROI 2;(d)-(h); GVF fields and of segments.

**Figure 5:**
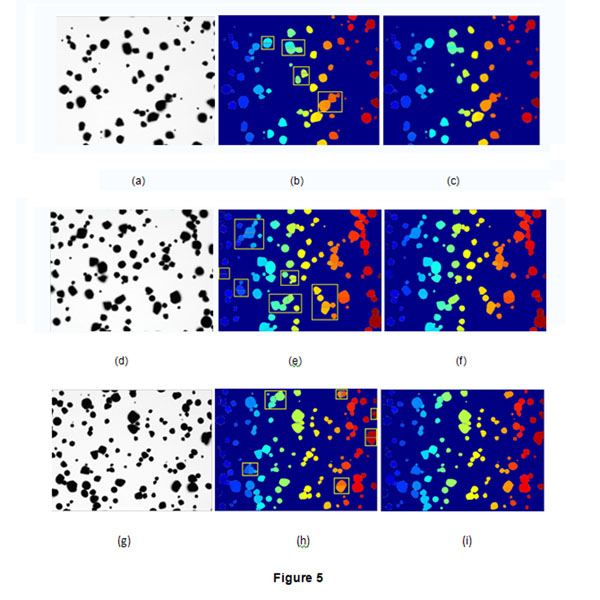
**Results of three images by the proposed method.** Three big images are processed by the proposed method. (a),(d) and (g) are the original images, (b),(e) and (h) are the results obtained by watershed algorithm, 5(c),5(f) and 5(i) are the final results. It shows that the proposed method can effectively merge those oversegments in the rectangle regions.

To identify oversegmentation, we computed the roundness of all the segments obtained by watershed algorithm. Fig.3 and Table 1 are the roundness values of the segments in Fig.1 (e). Segments 1,3,4,5,6,11,13 have roundness values less than 0.70 and they are oversegments (parts of an object) except for segment 1(background), while segments 2,7,8,9,10,12,14,15 have roundness values more than 0.70 and these segments should be further determined if they are oversegments using critical point criterion. Therefore, we tried to search the critical points in the neighbourhood of the centre of a segment with a big roundness value using GVF field analysis. For convenience, we use two ROIs including oversegments in Fig. 1(e) to discuss. The two ROIs selected from Fig. 1(e) are shown in Fig. 4 (a). Fig.4 (b) and Fig.4(c) are the enlarged versions of ROI 1 and ROI 2 respectively. Fig.4(d) shows the GVF field of segments 2,3,4,5 and 6, Fig.4(e),5(f) and 5(g) are the GVF fields of segments 7,8 and 9, Fig.4(h) is the GVF fields of segments 10,11,12,13,14,15. In each segment, the circle filled with colour represents the segment centre and the diamond filled with the same colour is the critical point of the segment. In order to differentiate the centres and critical points in different segments, different segments adopt different colours. From the GVF fields in Fig.4 (d)-5(h) and the critical points searching results in Table 1, we can know that the proposed search method found the critical points in the neighbourhood of the centres of the segments 2,7,8,9,10,14,15. Although the roundness of segment 12 is more than 0.70, there is not critical point in the neighbourhood of the segment centre. Thus it’s an oversegment and should be merged into a core segment.

**Table 1:** Roundness values and the critical points found in the GVF fields.

Segment no	1	2	3	4	5	6	7	8	9	10	11	12	13	14	15
Roundness	0.654	0.761	0.16	0.352	0.26	0.26	0.911	0.785	0.877	0.771	0.357	*0.748*	0.208	0.749	0.907

Critical point		√					√	√	√	√				√	√

Tick denotes the critical point was found, the single objects are bold, segment 12 is an oversegment because there is no critical point inside its GVF fields

From the roundness and critical points of the segments, we can conclude there are 7 starch granules (they are segments 2, 7, 8, 9, 10, 14, 15), and other segments except for segment 1(background) should be merged. Fig.4 (f) is the final result after merging by the FCM. In ROI 1, segments 3, 4 and 5 were merged into segment 2, while segment 6 was merged into segment 8; in ROI 2, segments 11 and 13 were merged into segment 10, while segment 12 was merged into segment 14.

In order to testify the proposed algorithm, 20 starch images were processed by the proposed method. Fig.5 shows three samples of the twenty images and the segmentation results. Fig. 5(a),5(d) and 5(g) are the original image, Fig.5(b), 5(e) and Fig.5(h) are the results obtained by watershed algorithm. From the figures, we can find that there exist many oversegments in the rectangle regions. Fig.5(c) and 5(f) and 5(i) show the results after the proposed method was used to merge those segments effectively.

## Discussion

From the intensity distribution of microscopic images of starch granules, we can know there are two peaks corresponding to starch granules and background respectively, therefore, we investigated automatic thresholding to separate objects from background. The threshold was initialized by the middle of intensity range and adjusted by the difference between previous threshold and the mean of the intensity of pixels belong to the two classes during an iterative process. After the binary image was acquired, we can calculate its Chamfer distance and then segment roughly starch granules using watershed method. Since the shapes of most of the starch granules are round or nearly round, we computed roundness so as to automatically identify the oversegment. In our experiments, it is good if the threshold of roundness is from 0.7 to 0.75. However, some single starch granules have roundness less than the experimental threshold and some oversegments have big roundness. In order to determine the real single object and oversegments effectively, the GVF field is applied to search the critical points in the neighbourhood of an segment center. It can be considered as a single object if it has big roundness value and a critical point around its center, or else it’s an oversegment. Therefore, these two criteria such as roundness and critical point can be used to determine the object number automatically. Fuzzy c-means clustering is a powerful technique to merge adjacent segments with similar features. How to define features which can represent a starch granule effectively is a key issue. Since intensity and position are the primary characteristics of a segment, we adopted features including mean intensity, intensity variance and distance between the centers of the ROI and the segment. Different weights were assigned to these features so as to reveal its significance, center distance is given bigger weight because only the adjacent segments may be merged.

## Conclusions

We proposed a novel method to segment microscopic images of starch granules. In order to deal with the oversegmentation of watershed algorithm, we calculated the roundness of the segments and analyzed the GVF field so as to identify the segments and determine core segment automatically. Position and intensity of segments were extracted as input features for fuzzy c-means clustering. Experimental results show that the oversegments could be merged successfully and the proposed algorithm may obtain prominent performance in object segmentation.

## Competing interests

The authors declare that they have no competing interests.

## Authors' contributions

SG and JT developed the algorithm and wrote the paper. QX acquired the images and helped writing paper. YD helped writing the paper. All authors read and approved the final manuscript.
